# Academic and psychological determinants of mental stress among medical students in Dhaka: A multinomial logistic regression approach

**DOI:** 10.1371/journal.pone.0354261

**Published:** 2026-07-20

**Authors:** Maliha Khan Majlish, Mushfiqur Rahman Khan Majlish, Sabrin Shohid, Md Samiul Islam, A. N. M. Shamsul Islam

**Affiliations:** 1 Department of Public Health and Hospital Administration, National Institute of Preventive and Social Medicine (NIPSOM), Mohakhali, Dhaka, Bangladesh; 2 Department of Statistics, University of Dhaka, Dhaka, Bangladesh; 3 Department of Medicine, University Hospitals of Leicester NHS Trust, Leicester, Leicestershire, England; King Abdulaziz University Faculty of Medicine, SAUDI ARABIA

## Abstract

As stress among medical students is a global health concern due to comprehensive academic engagements, this study aimed to assess the educational and psychological factors related to mental stress among medical students in Dhaka city. A descriptive type of cross-sectional study was conducted in two medical colleges in Dhaka city among 380 medical students from 3^rd^ year to 5^th^ year, using a pre-tested, self-administered, and structured questionnaire. The data obtained were analyzed using IBM SPSS Statistics (version 23) and Stata (version 14.2) employing the chi-square association test for bivariate analysis and subsequent multinomial logistic regression for the significant variables. Among 380 respondents, 68.7% were female, with 51.8% of respondents from Shaheed Suhrawardy Medical College. The mean stress score of medical students was 21.64 (SD = 6.64), with 67.9% and 22.6% as moderately and highly stressed, respectively. In bivariate analysis, 29 academic factors and 09 psychological factors were significantly associated with stress. In multinomial logistic regression analysis, three models were conducted, containing variables related to academic stress in model 1, variables related to psychological stress in model 2, and all stress variables in model 3. From model 1, academic performance, study and exam score anxiety, fear of future uncertainty and new living, administration satisfaction, and lack of entertainment facilities are significantly associated with stress. Additionally, the factors significantly associated with stress are time management, social life perception, and feeling lonely for model 2 and all stress-related variables, including study and exam score worry, difficulty in understanding context, fear of new living, administration and lecture satisfaction, teaching strategy satisfaction, time management, parental support in study, and feeling loneliness for model 3. Proper interventions for stressors of mental stress, further assessment, and rehabilitation for high and moderate-stressed respondents could be a prime concern to lessen the burden of stress among medical students.

## Introduction

Despite modern advancements, mental disorders persist as a major global health crisis. Stress, triggered by various situations when powerless or unable to achieve desired outcomes, is the body’s response to mental strain, releasing stress hormones and activating the defense system. However, this response can have both positive and negative effects on mental and physical health. Global statistics indicate 61% of adults under stress feel nervous, and 51% feel depressed [1]. Developed countries like the Netherlands (depression, anxiety: 20%, 17%), Saudi Arabia (stress: 33.8%), Argentina, Canada, and the USA show significant but lower ranges of mental health problems, with medical students facing higher distress than the general population [2–5]. Being widespread among medical students globally, anxiety and depression rates are high in the Middle East and Asia. In Southeast Asia, Pakistan (depression in 57.6%, anxiety in 74%, and stress in 57.7% of students) [6], India (depression 8.7% to 71.3%, anxiety 34.5%, and stress 51.3%) [7], Nepal (20.9% of psychological morbidity) [8], China (depression, anxiety, stress: 12.26%, 18.47%, 8.53%), Turkey (depression, anxiety, stress: 18.6%, 26.5%, 7.9%), Egypt (depression, anxiety, stress: 64.2%, 77.1%, 70.4%), and Malaysia (stress: 33.3%) reveal significant prevalence rates of anxiety, depression, and stress among students and medical professionals [9–12,48], emphasizing the global concern for mental health, regardless of cultural or socio-demographic differences [2–5,7–13] Moreover, Bangladesh ranks 153rd globally in suicide rates, with approximately 7 million people diagnosed with anxiety and depression, accounting for 0.84% of all fatalities [14,15]. Depression (57.9%), anxiety (33.7%), stress (59.7%), and sleep disorders are prevalent in Bangladesh [16], with medical students experiencing higher stress (54.6%), anxiety (65.9%), and depression (49.9%), primarily due to studies [17,18].

Bangladesh has 106 medical colleges, including 36 public, numerous private, and 6 under the Ministry of Defense, collectively enrolling over 10,000 students annually [22]. The MBBS program spans five years, followed by a mandatory one-year internship, with multiple phases of examinations that place substantial academic pressure on students. Stress affects 54% of medical students in Bangladesh, while the percentages of stress in medical students are comparable with respect to sex. Key stressors include workload, time constraints, family expectations, lack of practical training, academic pressures, and fear of entering the medical field, etc., which are all major contributors to mental health issues [18,23–25]. Lifestyle factors such as excessive screen time, physical inactivity, poor sleep, irregular meal frequency, and alcohol consumption are closely linked to depression and anxiety [20]. While some students develop coping strategies in their middle years, the cumulative demands of medical school continue to heighten psychological vulnerability [26,27].

Given that depression, stress, and anxiety among medical students are well-documented globally [18–21,23–25], understanding their prevalence and underlying causes in Bangladesh is imperative, as evidence for academic and psychological stressors remains limited, fragmented, and often focused on single institutions or small student groups. Few studies have systematically assessed perceived stress using a validated tool, and limited exploration of academic and psychological stressors specific to the country’s unique social context hampers policymakers and educators from designing targeted mental health interventions for medical students. This study aimed to identify the academic and psychological determinants of mental stress among medical students in Dhaka using the Perceived Stress Scale (PSS) and a multinomial logistic regression approach. Specifically, the objectives were to (i) measure the prevalence of perceived stress, (ii) examine the key academic and psychological factors associated with varying levels of stress, and (iii) generate evidence to inform policies and interventions that promote mental health and wellbeing among medical students.

## Methodology

### Study design and setting

A descriptive cross-sectional study was conducted among medical students in Dhaka to evaluate their mental stress and associated factors related to academic activities from January 1 to December 31, 2022, with data collection period from September 3 to September 21, 2022. Two medical colleges, such as one government medical college as Shaheed Suhrawardy Medical College & Hospital, and one private medical college as Shaheed Monsur Ali Medical College and Hospital, around Dhaka city, were selected through stratified sampling techniques to ensure representation from both public and private institutions. The study population included students from 3^rd^, 4^th^, and 5^th^ academic years in these institutions, as students of this group are simultaneously engaged in intensive academic coursework, ward duties, and examinations. Moreover, the well-documented academic and psychological burden faced by Bangladeshi medical students has been established during their clinical years [24,28,29], creating a scenario where stress is both highly prevalent and diverse in its manifestations, making this group suitable for the study objectives.

### Sample size and sampling technique

The required sample size was determined using the formula $n=\frac{z^{\text{2}}pq}{d^{\text{2}}}$, where *z* = 1.96 (standard normal deviate at 95% confidence level), *p* = 0.50 (assumed prevalence of stress among medical students based on previous studies in Bangladesh to maximize sample size), *q* = 1–p, and *d* = 0.05 (margin of error). Based on this calculation, the minimum required sample size was 345. With a 10% non-response rate, the final target sample size was 380 students.

At the time of data collection, the total number of 3^rd^–5^th^ year students was 618 across the two selected medical colleges (Shaheed Suhrawardy Medical College: 319 students; Shaheed Monsur Ali Medical College: 299 students). A stratified random sampling method was employed to ensure representation from both public and private medical institutions in Dhaka. i) Stratification: All medical colleges in Dhaka were divided into two strata based on type: public medical colleges and private medical colleges. ii) Random selection: One medical college from each stratum was selected using simple random sampling, resulting in Shaheed Suhrawardy Medical College (public) and Shaheed Monsur Ali Medical College (private). iii) Census within selected colleges: All eligible 3^rd^, 4^th^, and 5^th^-year students who provided informed consent in the selected medical colleges were invited to participate, exclusion criteria ruled out those who did not consent or were unable to participate due to severe illness or acute psychological distress.

### Pre-testing

A pre-test was conducted at Bangladesh Medical College & Hospital using the same structured self-administered questionnaire. A total of 55 students were recruited after authorization from the college principal. Self-administered structured questionnaires were distributed and collected four days later, with an additional two days allowed if necessary. Pre-test results showed a response rate of 72.7% (n = 40). Based on the pre-test results, minor modifications were made to improve clarity and flow of the questions, including rewording ambiguous items and adjusting the sequence for better logical order. The revised questionnaire was then used at the study sites.

### Data collection procedures

**Fig 1:**
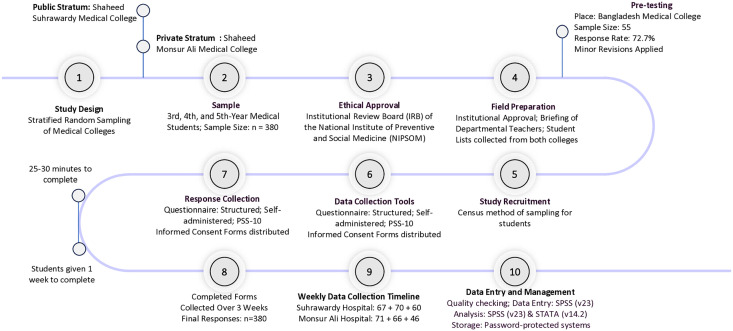
Flow of study data collection procedures.

Institutional permissions were obtained, and departmental teachers facilitated student briefings. A pre-tested, structured, self-administered questionnaire and informed consent form were distributed. On average, the questionnaire comprised four sections and required 25–30 minutes to complete. The study team accounted for real-life challenges faced by medical students during data collection; for example, many students were on ward rotations or preparing for examinations. To minimize disruption, the questionnaire distribution was integrated into existing departmental briefings, with students allowed flexibility in returning responses within a week. Teachers also played a supportive role by encouraging participation while ensuring voluntary and anonymous involvement. Completed responses were collected over three weeks, yielding a total of 380 valid responses (Fig 1).

## Study materials

### Semi-structured questionnaire

The questionnaire was created in both English and Bengali, with the English version distributed to respondents. It consisted of four sections covering: Socio-demographic characteristics, Academic activities-related questions, Factors associated with mental stress, and Perceived Stress Scale (PSS).

### Independent and dependent variables.

The dependent variable comprised the mental stress score utilizing the Perceived Stress Scale-10, while the independent variables comprised the following:

*Sociodemographic variables*: Respondent age group, sex, Medical College name, religion, nationality, academic year, marital status, family type, place of residence, accommodation, parents’ occupation, whether parents bear educational expense, whether the student has a part-time job, etc.

*Academic activities related variables*: Academic performance, attending sessions, concentration in sessions, understanding sessions, perception of academic sessions curriculum difficulty, academic environment learning material availability, study and exam score anxiety, pressure of studies and passing exams, time management pressure, difficulty in understanding context, parent’s and teacher’s expectation pressure, fear of future uncertainty and new living, fear of dropping out of course, future choice cluelessness, administration satisfaction, lecture satisfaction, exam criteria satisfaction, career choice satisfaction, lack of entertainment facility, teaching strategy satisfaction, course activity feeling, interaction with other medical students, recreation time

*Psychological factors*: Time management skills, parental support in study, social life perception, feeling lonely, studies hampering social interaction, illness hampering study, classmate conflict, family conflict, roommate conflict.

### Perceived stress scale-10

Though several scales are available to assess stress among medical students, the Perceived Stress Scale (PSS-10) is widely validated, brief, and easily adaptable to local languages, making it suitable for cross-cultural studies. In contrast, other widely validated tools measure depression, anxiety, and stress [30–32] simultaneously, but are comprehensive with less focus on perceived stress alone. Other self-reported questionnaires can target specific stressors but often require cultural adaptation and validation. Considering these factors, the PSS-10 was selected for this study due to its brevity, ease of administration, and suitability for assessing perceived stress in Bangladeshi medical students.

The PSS-10, developed by Cohen et al. (1983), is a widely validated psychological tool used to assess perceived stress over the past month, evaluating how unpredictable, uncontrollable, and overwhelming respondents find their lives. It consists of 10 items on a 5-point Likert scale (0 = never to 4 = very often), with items 4, 5, 7, and 8 being reverse-coded (0 = 4, 1 = 3, 2 = 2, 3 = 1, 4 = 0), and the sum of all items yields a total score ranging from 0 to 40. Stress levels are classified as: Low stress (0−13 scores); Moderate stress (14−26 scores); High Stress (27−40 scores) (33). To ensure better comprehension, the questionnaire was administered in both English and Bangla, using a validated Bangla version of the PSS-10 [34].

Among 380 medical students surveyed, responses were obtained using a 10-item PSS scale, administered in both English [33,35] and Bangla [34,36] version of the English self-administered questionnaire for better comprehension.

### Data management

#### Data pre-processing.

At the end of each day of data collection, each questionnaire was reviewed for completeness and consistency, and data entry commenced immediately afterward. Once all data were collected, they were processed and tabulated, involving careful editing, coding, recoding, summation, and categorization to exclude irrelevant and unreliable information. The missing value count was low (less than 5%). The missing value count was low (less than 5%) and was addressed using mode imputation.

#### Data analysis.

The Chi-square association test was conducted for bivariate analysis to identify the unadjusted significance between the dependent and independent variables without controlling for other factors. Predictors with a p-value <0.10 in bivariate analysis were considered in the regression model. Multinomial logistic regression was employed to identify the factors associated with varying levels of stress (low, moderate, and high) among medical students. The study included three models: Model 1 comprised variables related to academic stress, Model 2 comprised variables related to psychological stress, and Model 3 included all stress-related variables. The results are presented as Relative Risk Ratios (RRRs) with corresponding p-values, indicating the likelihood of being in the moderate or high stress group compared to the low stress reference group, given the presence of specific predictors. All statistical tests were two-sided, and the significance level was set at 10%. IBM SPSS Statistics (version 23) and STATA version 14.2 were used to conduct statistical analysis.

### Ethical considerations

Regarding ethical implications, the protocol was approved by the Institutional Review Board (IRB) of the National Institute of Preventive and Social Medicine (NIPSOM), and permissions from the medical colleges were obtained. Informed written consent was obtained from each respondent, and the study objectives were briefly explained to them. Privacy and confidentiality were strictly maintained, and no personal identifiers were collected. Participants had the right to withdraw from the study at any time and were assured that there would be no physical or mental harm, as no invasive procedures were involved.

## Results

### Socio-demographic factors

**Fig 2:**
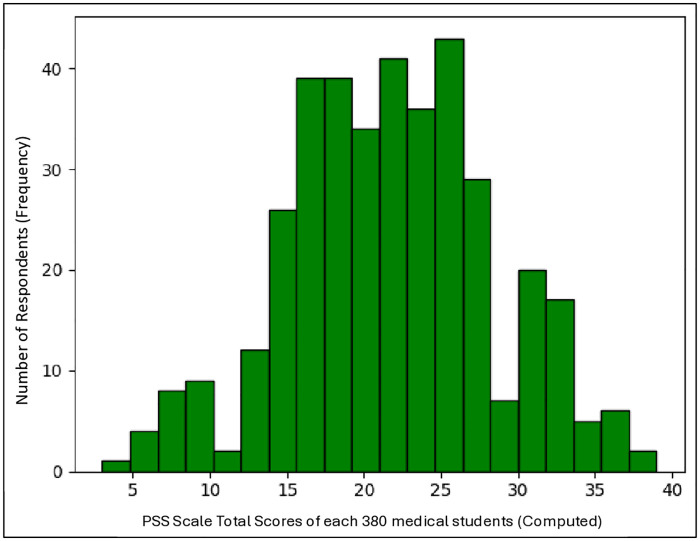
Histogram of PSS-10 score among the among medical students (n = 380).

In this study, Fig 2 shows the average PSS-10 score was 21.64 with a standard deviation of 6.64 (minimum: 3 and maximum: 39). 258 respondents (67.9%) had a moderate stress level among all 380 respondents. The perceived stress scores range between 15–27.

Among 380 respondents, 96.6% of respondents were aged between 21–25, and 68.7% of respondents were female. 51.8% of respondents were from Shaheed Suhrawardy Medical College, and the remaining from Shaheed Monsur Ali Medical College. 86.8% belong to the Islamic religion, and 15.8% of respondents were foreign nationals. Almost an equal number of respondents from different academic years participated in the study. 92.4% were unmarried respondents, and 83.9% came from nuclear families. 71.3% resided in urban residences, and 62.9% lived in hostels. In case of parents’ occupation, 38.2% of respondents’ fathers worked in government sectors, whereas 69.7% of respondents’ mothers were homemakers. 88.2% of respondents’ parents bear their educational expenses, and 74.2% of students did not have a part-time job.

### Academic factors

Table 1 shows the academic factors that are significantly associated with perceived stress in the study sample at the 10% significance level. Almost an equal number of respondents have different perceptions of their academic performance (satisfactory or not satisfactory). 91.1% of students attended academic sessions regularly, and 69.5% gave concentration in these sessions. 66.3% understood the studies delivered in these sessions, and 78.4% found these sessions beneficial. 73.9% of students found the medical curriculum difficult. Almost an equal number of students were either satisfied or dissatisfied with the academic environment, and 68.2% found the availability of learning materials. 84.7% of respondents worry about studying, and 74.7% worry about poor exam scores. Almost an equal number of students felt the pressure of studying, passing exams, and time management in studies (93.9%, 93.4% and 91.8%). 63.7% of respondents felt difficulty in understanding context, and 95.3% felt the vastness of the academic syllabus. 62.9% felt the pressure of fulfilling parents’ expectations, and 68.4% felt the pressure of fulfilling teachers’ expectations. 79.7%, 66.7% and 56.1% of students were fearful of future uncertainty, new way of living, and dropping out of the course, respectively. 67.4% of respondents were clueless about future choices of specialty. 31.6% were satisfied with the administration, and 35.5% were satisfied with the examination criteria. An equal number of students were satisfied with class lectures and career choice (54.5% and 56.5% respectively). 41.3% were satisfied with teaching strategies, and 42.4% were satisfied with course activities. 59.7% of respondents had a satisfactory interaction with other medical students. 81.3% of students felt the lack of entertainment facilities in institutions, and 82.1% of students had no time for recreation.

**Table 1:** Academic factors associated with perceived stress (N = 380).

Variable		Number of Observations (%)	P value*
Academic Performance	Satisfactory	186 (48.9%)	0.000
	Not Satisfactory	194 (51.1%)	
Attending Sessions	Yes	346 (91.1%)	0.002
	No	34 (8.9%)	
Concentration in Sessions	Yes	264 (69.5%)	0.000
	No	116 (30.5%)	
Understanding Sessions	Yes	252 (66.3%)	0.000
	No	128 (33.7%)	
Perception of Benefit of Academic Sessions	Beneficial	298 (78.4%)	0.069
	Not Beneficial	82 (21.6%)	
Curriculum Difficult	Yes	281 (73.9%)	0.000
	No	99 (26.1%)	
Academic Environment	Satisfactory	187 (49.3%)	0.001
	Not Satisfactory	192 (50.5%)	
Learning Material Availability	Available	259 (68.2%)	0.066^a^
	Not Available	121 (31.8%)	
Study Worry	Yes	322 (84.7%)	0.000
	No	58 (15.3%)	
Exam Score Worry	Yes	284 (74.7%)	0.000
	No	96 (25.3%)	
Pressure of Studies	Yes	357 (93.9%)	0.010
	No	23 (6.1%)	
Pressure of Passing Exams	Yes	355 (93.4%)	0.000
	No	25 (6.6%)	
Time Management Pressure	Yes	349 (91.8%)	0.001
	No	31 (8.2%)	
Difficulty in Understanding Context	Yes	242 (63.7%)	0.000
	No	138 (36.3%)	
Parent’s Expectation Pressure	Yes	239 (62.9%)	0.011
	No	141 (37.1%)	
Teacher’s Expectation Pressure	Yes	260 (68.4%)	0.003
	No	120 (31.6%)	
Future Uncertainty Fear	Yes	303 (79.7%)	0.000
	No	77 (20.3%)	
Fear of New Living	Yes	254 (66.8%)	0.000
	No	126 (33.2%)	
Fear of Dropping Out of Course	Yes	215 (56.6%)	0.000
	No	165 (43.4%)	
Future Choice Cluelessness	Yes	256 (67.4%)	0.000
	No	124 (32.6%)	
Administration Satisfaction	Yes	119 (31.3%)	0.000
	No	261 (68.7%)	
Lecture Satisfaction	Yes	207 (54.5%)	0.000
	No	173 (45.5%)	
Exam Criteria Satisfaction	Yes	135 (35.5%)	0.000
	No	245 (64.5%)	
Career Choice Satisfaction	Yes	215 (56.6%)	0.000
	No	165 (43.4%)	
Lack of Entertainment Facility	Yes	309 (81.3%)	0.003
	No	71 (18.7%)	
Teaching Strategy Satisfaction	Yes	157 (41.3%)	0.032
	No	233 (58.7%)	
Course Activity Feeling	Satisfactory	161 (42.4%)	0.000
	Not Satisfactory	219 (57.6%)	
Medical Students Interaction	Satisfactory	227 (59.7%)	0.023
	Not Satisfactory	153 (40.3%)	
Recreation Time	Yes	68 (17.9%)	0.000
	No	312 (82.1%)	

In * column, significant when Confidence Interval (CI) 90%, p < 0.1.

Among academic factors, 29 variables namely perception of academic performance, attending academic session, concentration in academic sessions, understanding academic sessions, perception of benefit of academic sessions, finding medical curriculum difficult, satisfaction with academic environment, availability of learning material, worry about study, worry about exam score, pressure of studies, pressure of passing exams, pressure of time management, difficulty in understanding context, pressure of parents’ expectations, pressure of teachers’ expectations, fear of future uncertainty, fear of new living, fear of dropping out of course, cluelessness of future choice, administration satisfaction, lecture satisfaction, exam criteria satisfaction, career choice satisfaction, lack of entertainment facility, satisfaction in teaching strategy, feeling about course activity, interaction with medical students’ and recreation time were significantly associated at the 10% significance level. The remaining academic factors are insignificant at the 10% level of significance.

### Psychological factors

Table 2 exhibits the other factors that are significantly associated with perceived stress in the study sample at 5% significance level. Among 380 medical students, 27.6% were satisfied with their time management skills, and 86.3% had parental support in study. 35.3% of the students were satisfied with their current social life, and 66.8% were suffering from loneliness. 68.4% felt that studies hamper social interaction, and 53.2% felt that illness hampers study. 18.9% of students are engaged in conflicts with their family members, and an equal number of students are involved in conflict with roommates and classmates (23.7%).

**Table 2:** Psychological factors associated with perceived stress (N = 380).

Variable		Number of Observations (%)	P value*
Time Management Skill	Satisfactory	105 (27.6%)	0.000
	Not Satisfactory	275 (72.4%)	
Parental Support in Study	Present	328 (86.3%)	0.041
	Absent	52 (13.7%)	
Social Life Perception	Satisfactory	134 (35.3%)	0.000
	Not Satisfactory	246 (64.7%)	
Feeling Lonely	Yes	254 (66.8%)	0.000
	No	126 (33.2%)	
Studies Hamper Social Interaction	Yes	260 (68.4%)	0.021
	No	120 (31.6%)	
Illness Hampers Study	Yes	202 (53.2%)	0.005
	No	178 (46.8%)	
Classmate Conflict	Yes	90 (23.7%)	0.042
	No	290 (76.3%)	
Family Conflict	No	308 (81.1%)	0.012
	Yes	72 (18.9%)	
Roommate Conflict	No	290 (76.3%)	0.008
	Yes	90 (23.7%)	

In * column, all values are significant when Confidence Interval (CI) 95%; p < 0.10.

Among psychological factors, 09 variables, namely time management skill, parental support in study, perception of social life, feeling lonely, studies hamper social interaction, illness hampers study, classmate conflict, family conflict, and roommate conflict, were significantly associated at the 10% significance level, respectively. The remaining factors are insignificant at the 10% level of significance.

### Multivariate analysis

#### Academic factors.

Table 3 exhibits the relative risk ratio (RRR) for various academic factors on Perceived Stress and their corresponding significance among participating medical students. RRR and significance values for each academic factor are measured for moderate and high perceived stress. From the p-value, it is observed that anxiety regarding exam scores and satisfaction with administration are significantly associated with moderate stress of the students at the 10% level of significance. The RRR value is 0.392 for anxiety related to exam scores, which means that students who do not worry about their exam scores are 60.8% less likely to experience moderate stress compared to those who do worry. Moreover, the RRR value is 3.598 for administration satisfaction, which means that students who are not satisfied with the administration system are 3.598 times more likely to experience moderate stress compared to those who are satisfied with the administration. The remaining academic factors are insignificant at 5% level of significance.

**Table 3:** Relative risk ratio of academic factors on perceived stress (N = 380).

Variable	Category	RRR (moderate)	95% CI	P value (moderate)	RRR (high)	P value (high)	95% CI
Academic Performance	Satisfactory	1		–	1	–	
	Unsatisfactory	2.265	(0.703, 7.331)	0.172	2.979	0.099a	(0.815, 10.883)
Attending Sessions	Yes	1		–	1	–	
	No	0.443	(0.507, 3.403)	0.434	1.054	0.962	(0.120, 9.233)
Concentration in Sessions	Yes	1		–	1	–	
	No	0.751	(0.192, 2.941)	0.682	1.051	0.947	(0.241, 4.584)
Understanding Sessions	Yes	1		–	1	–	
	No	1.103	(0.308, 3.945)	0.879	1.078	0.308	(0.509, 8.490)
Perception of Sessions	Beneficial	1		–	1	–	
	Not Beneficial	1.331	(0.311, 5.683)	0.699	1.106	0.901	(0.226, 5.394)
Curriculum Difficulty	Yes	1		–	1	–	
	No	0.760	(0.281, 2.054)	0.589	0.786	0.712	(0.219, 2.814)
Academic Environment	Satisfactory	1		–	1	–	
	Unsatisfactory	0.767	(0.221, 2.656)	0.676	0.589	0.460	(0.145, 2.397)
Learning Material Availability	Available	1		–	1	–	
	Not Available	0.580	(0.174, 1.935)	0.376	0.528	0.354	(0.137, 2.037)
Study Worry	Yes	1		–	1	–	
	No	0.510	(0.162, 1.603)	0.250	0.140	0.085a	(0.015, 1.308)
Exam Score Worry	Yes	1		–	1	–	
	No	0.392	(0.144, 1.064)	0.066a	0.216	0.028b	(0.055, 0.846)
Pressure of Studies	Yes	1		–	1	–	
	No	0.982	(0.169, 5.684)	0.984	1.156	0.919	(0.071, 18.891)
Pressure of Passing Exams	Yes	1		–	1	–	
	No	0.328	(0.067, 1.608)	0.170	1.123	0.928	(0.091, 13.935)
Time Management Pressure	Yes	1		–	1	–	
	No	1.259	(0.293, 5.396)	0.756	0.406	0.508	(0.028, 5.858)
Difficulty in Understanding Context	Yes	1		–	1	–	
	No	1.669	(0.591, 4.715)	0.333	1.489	0.523	(0.438, 5.062)
Parent’s Expectation Pressure	Yes	1		–	1	–	
	No	1.649	(0.518, 5.247)	0.397	1.926	0.334	(0.508, 7.298)
Teacher’s Expectation Pressure	Yes	1		–	1	–	
	No	1.020	(0.337, 3.086)	0.972	1.061	0.930	(0.280, 4.020)
Fear of Future Uncertainty	Yes	1		–	1	–	
	No	0.593	(0.171, 2.049)	0.409	0.133	0.050a	(0.018, 0.998)
Fear of New Living	Yes	1		–	1	–	
	No	0.412	(0.134, 1.266)	0.122	0.281	0.062a	(0.074, 1.064)
Fear of Dropping Out of Course	Yes	1		–	1	–	
	No	0.776	(0.264, 2.277)	0.645	0.537	0.318	(0.158, 1.819)
Future Choice Cluelessness	Yes	1		–	1	–	
	No	0.686	(0.267, 0.176)	0.435	0.904	0.865	(0.282, 2.892)
Administration Satisfaction	Yes	1		–	1	–	
	No	3.598	(1.204, 10.785)	0.022b	3.770	0.045b	(1.031, 13.779)
Lecture Satisfaction	Yes	1		–	1	–	
	No	1.743	(0.454, 6.695)	0.418	3.371	0.112	(0.753, 15.089)
Exam Criteria Satisfaction	Yes	1		–	1	–	
	No	0.670	(0.184, 2.433)	0.543	0.901	0.892	(0.202, 4.022)
Career Choice Satisfaction	Yes	1		–	1	–	
	No	1.096	(0.351, 3.417)	0.874	1.762	0.382	(0.494, 6.284)
Lack of Entertainment Facility	Yes	1		–	1	–	
	No	0.490	(0.184, 1.303)	0.153	0.273	0.060a	(0.071, 1.054)
Teaching Strategy Satisfaction	Yes	1		–	1	–	
	No	1.059	(0.292, 3.843)	0.930	0.317	0.130	(0.071, 1.399)
Course Activity Feeling	Satisfactory	1		–	1	–	
	Unsatisfactory	0.480	(0.138, 1.662)	0.247	0.740	0.685	(0.173, 3.157)
Medical Students’ Interaction	Satisfactory	1		–	1	–	
	Unsatisfactory	1.924	(0.638, 5.801)	0.245	1.775	0.359	(0.521, 6.053)
Recreation Time	Yes	1		–	1	–	
	No	0.858	(0.306, 2.405)	0.772	2.760	0.184	(0.618, 12.331)

a significant when Confidence Interval (CI) 90%, p < 0.1.

b significant when Confidence Interval (CI) 95%; p < 0.05.

In case of association with high stress, perception of academic performance, anxiety related to study and exams, fear of future uncertainty and new living, administration satisfaction, and lack of entertainment facilities are significantly associated with perceived stress at the 10% level of significance. From RRR, students who are not satisfied with their academic performance are 2.979 times higher relative risk to experience high stress than low stress compared to those who are satisfied with their academic performance. Anxiety related to study and exams is also significantly associated with high stress. Students who were not worried about their studies had an 86.0% lower relative risk of being in the high stress group rather than the low stress group compared with students who were worried about their studies (RRR = 0.140). Similarly, students who do not worry about their exams have a 78.4% lower relative risk of experiencing stress than those who worry about exam scores. Students who are not fearful of future uncertainty had an 86.7% lower relative risk of experiencing stress than those who are uncertain about their future. Additionally, students who are not fearful of new living have a 71.9% lower relative risk of experiencing high stress compared to those who are fearful of new living. Similarly, moderate stress and administration satisfaction are also significantly associated with high perceived stress. Students who are not satisfied with the administration have a 3.77 times higher relative risk of experiencing high stress than those who are satisfied. Lack of entertainment facilities is also associated with high perceived stress, and students who reported no lack of entertainment facilities had a 72.7% lower relative risk of being in the high stress group rather than the low stress group compared with students who reported a lack of entertainment facilities (RRR = 0.273). The remaining variables are not significant at the 10% level of significance.

### Psychological factors

Table 4 exhibits the relative risk ratio (RRR) for various psychological factors on Perceived Stress and their corresponding significance among participating medical students. RRR and significance values for each psychological factor are measured for moderate and high perceived stress. From the p-value, it is observed that perception of time management and feeling of loneliness are significantly associated with both moderate and high perceived stress, and perception of social life is significantly associated with high perceived stress at 5% level of significance.

**Table 4:** Relative risk ratio of psychological factors on perceived stress (N = 380).

Variable	Category	RRR (moderate)	95% CI	P value (moderate)	RRR (high)	95% CI	P value (high)
Time Management	Satisfactory	1		–	1		–
	Unsatisfactory	4.819	(2.0845, 11.144)	0.000^a^	13.827	(4.451, 42.946)	0.000^a^
Parental Support in Study	Present	1		–	1		–
	Absent	2.565	(0.316, 20.825)	0.378	3.117	(0.356, 27.235)	0.304
Social Life Perception	Satisfactory	1		–	1		–
	Unsatisfactory	1.194	(0.517, 2.759)	0.677	4.146	(1.409, 12.201)	0.010^a^
Feeling Lonely	Yes	1		–	1		–
	No	0.278	(0.113, 0.685)	0.005^a^	0.105	(0.034, 0.325)	0.000^a^
Studies Hamper Social Interaction	Yes	1		–	1		–
	No	0.843	(0.369, 1.926)	0.686	1.082	(0.396, 2.956)	0.876
Illness Hampers Study	Yes	1		–	1		–
	No	1.097	(0.471, 2.556)	0.829	0.804	(0.301, 2.153)	0.666
Classmate Conflict	No	1		–	1		–
	Yes	1.112	(0.316, 3.912)	0.868	2.774	(0.712, 10.807)	0.141
Family Conflict	No	1		–	1		–
	Yes	1.973	(0.395, 9.839)	0.427	2.118	(0.388, 11.544)	0.386
Roommate Conflict	No	1		–	1		–
	Yes	1.775	(0.429, 7.337)	0.427	1.770	(0.387, 8.081)	0.461

^a^significant when Confidence Interval (CI) 95%; p < 0.05.

From the table, students who are not satisfied with their time management are 4.819 times more likely to experience moderate stress and 13.827 times more likely to experience high stress compared to those who are satisfied with their time management. Moreover, students who are not suffering from loneliness are 72.2% less likely to experience moderate stress and 89.5% less likely to experience high stress compared to those who are suffering from loneliness. Students who are not satisfied with their social life are 4.146 times more likely to experience high stress compared to those who are satisfied with their social life. The remaining variables are insignificant at the 5% level of significance.

### Academic and psychological factors

Table 5 exhibits the relative risk ratio (RRR) for various academic and psychological factors on Perceived Stress and their corresponding significance among participating medical students. RRR and significant values for each academic and psychological factor are measured for moderate and high perceived stress. From the p-values, it is observed that students who are not worried about their exams are 66% less likely to experience moderate stress compared to those who worry. Moreover, students who face difficulty in understanding medical contexts are 4.117 times more likely to experience moderate stress compared to those who understand the context. Additionally, students who are not afraid of new living are 79.2% less likely to experience stress than those who are afraid of new living. Students who are not satisfied with their administration and lectures are 7.749 times and 5.042 times more likely to experience moderate stress compared to those who are satisfied with their administration and lectures. Furthermore, students who are not satisfied with their time management are 5.299 times more likely to experience high stress than those who are satisfied. Students who do not feel lonely are 86.7% less likely to experience moderate stress than those who are suffering from loneliness.

**Table 5:** Relative risk ratio of all factors on perceived stress (N = 380).

Variable	Category	RRR (moderate)	95% CI	P value (moderate)	RRR (high)	95% CI	P value (high)
Academic Performance	Satisfactory	1		–	1		–
	Unsatisfactory	1.151	(0.291, 4.539)	0.841	1.168	(0.257, 5.301)	0.840
Attending Sessions	Yes	1		–	1		–
	No	0.168	(0.015, 1.835)	0.144	0.367	(0.031, 4.481)	0.432
Concentration in Sessions	Yes	1		–	1		–
	No	0.451	(0.096, 2.118)	0.313	0.559	(0.105, 2.979)	0.496
Understanding Sessions	Yes	1		–	1		–
	No	1.032	(0.235, 4.527)	0.966	2.019	(0.401, 10.166)	0.394
Perception of Sessions	Beneficial	1		–	1		–
	Not Beneficial	0.981	(0.201, 4.804)	0.981	0.978	(0.171, 5.605)	0.980
Curriculum Difficulty	Yes	1		–	1		–
	No	0.690	(0.196, 2.421)	0.563	0.720	(0.159, 3.259)	0.671
Academic Environment	Satisfactory	1		–	1		–
	Unsatisfactory	0.998	(0.237, 4.209)	0.998	0.704	(0.139, 3.552)	0.671
Learning Material Availability	Available	1		–	1		–
	Not Available	0.558	(0.147, 2.111)	0.391	0.419	(0.094, 1.869)	0.255
Study Worry	Yes	1		–	1		–
	No	0.410	(0.104, 1.614)	0.202	0.132	(0.011, 1.531)	0.099^a^
Exam Score Worry	Yes	1		–	1		–
	No	0.340	(0.095, 1.216)	0.097^a^	0.125	(0.024, 0.646)	0.013^b^
Pressure of Studies	Yes	1		–	1		–
	No	1.115	(0.125, 9.931)	0.922	1.491	(0.064, 34.793)	0.803
Pressure of Passing Exams	Yes	1		–	1		–
	No	0.241	(0.033, 1.772)	0.163	1.698	(0.095, 30.245)	0.718
Time Management Pressure	Yes	1		–	1		–
	No	1.100	(0.225, 2.365)	0.906	0.156	(0.008, 2.966)	0.217
Difficulty in Understanding Context	Yes	1		–	1		–
	No	4.117	(1.021, 16.609)	0.047^b^	3.216	(0.674, 15.341)	0.143
Parent’s Expectation Pressure	Yes	1		–	1		–
	No	1.957	(0.441, 8.676)	0.377	2.145		0.369
Teacher’s Expectation Pressure	Yes	1		–	1	(0.406, 11.330)	–
	No	0.864	(0.235, 3.177)	0.826	0.801		0.778
Fear of Future Uncertainty	Yes	1		–	1	(0.107, 3.727)	–
	No	0.641	(0.137, 2.996)	0.572	0.157		0.112
Fear of New Living	Yes	1		–	1	(0.016, 1.539(	–
	No	0.208	(0.053, 0.809)	0.023^b^	0.155		0.020^b^
Fear of Dropping Out of Course	Yes	1		–	1	(0.032, 0.751)	–
	No	0.756	(0.189, 3.015)	0.692	0.468		0.328
Future Choice Cluelessness	Yes	1		–	1	(0.102, 2.138)	–
	No	0.480	(0.139, 1.657)	0.246	0.625		0.524
Administration Satisfaction	Yes	1		–	1	(0.148, 2.641)	–
	No	7.749	(1.894, 31.701)	0.004^b^	9.9479		0.006^b^
Lecture Satisfaction	Yes	1		–	1	(1.921, 46.766)	–
	No	5.042	(0.921, 27.598)	0.062^a^	10.056		0.014^b^
Exam Criteria Satisfaction	Yes	1		–	1	(1.605, 63.009)	–
	No	0.197	(0.037, 1.044)	0.156	0.291		0.192
Career Choice Satisfaction	Yes	1		–	1	(0.045, 1.855)	–
	No	1.275	(0.327, 4.965)	0.726	1.704		0.486
Lack of Entertainment Facility	Yes	1		–	1	(0.379, 7.644)	–
	No	0.571	(0.167, 1.954)	0.373	0.338		0.180
Teaching Strategy Satisfaction	Yes	1		–	1	(0.069, 1.651)	–
	No	1.051	(0.232, 4.741)	0.949	1.209		0.075^a^
Course Activity Feeling	Satisfactory	1		–	1	(0.037, 1.171)	–
	Unsatisfactory	0.243	(0.056, 1.044)	0.157	0.463		0.373
Medical Students’ Interaction	Satisfactory	1		–	1	(0.085, 2.515)	–
	Unsatisfactory	1.004	(0.277, 3.631)	0.994	0.716		0.642
Recreation Time	Yes	1		–	1	(0.175, 2.922)	–
	No	0.770	(0.242, 2.453)	0.659	2.197		0.354
Time Management	Satisfactory	1		–	1	(0.415, 11.626)	–
	Unsatisfactory	5.299	(1.536, 18.287)	0.008^b^	9.089		0.005^b^
Parental Support in Study	Present	1		–	1	(1.961, 42.145)	–
	Absent	10.045	(0.562, 179.491)	0.117	16.376		0.066^a^
Social Life Perception	Satisfactory	1		–	1	(0.831, 32.715)	–
	Unsatisfactory	0.488	(0.134, 1.781)	0.278	1.841		0.436
Feeling Lonely	Yes	1		–	1	(0.396, 8.567)	–
	No	0.133	(0.037, 0.472)	0.002^b^	0.046		0.000^b^
Studies Hamper Social Interaction	Yes	1		–	1	(0.011, 0.209)	–
	No	2.149	(0.642, 7.189)	0.214	4.470		0.140
Illness Hampers Study	Yes	1		–	1	(1.072, 18.642)	–
	No	2.123	(0.621, 7.247)	0.229	2.119		0.283
Classmate Conflict	No	1		–	1	(0.537, 8.352)	–
	Yes	1.341	(0.235, 7.637)	0.741	3.382		0.199
Family Conflict	No	1		–	1	(0.525, 21.751)	–
	Yes	5.652	(0.527, 60.604)	0.152	5.642		0.170
Roommate Conflict	No	1		–	1	(0.477, 66.732)	–
	Yes	2.450	(0.435, 13.783)	0.309	2.034		0.456

a significant when Confidence Interval (CI) 90%, p < 0.1.

b significant when Confidence Interval (CI) 95%; p < 0.05.

In case of high stress, students who are not worried about their studies and exams are 86.8% and 87.5% less likely to experience high stress than those who are worried. Moreover, students who are not afraid of new living are 84.5% less likely to experience high stress compared to those who are afraid of new living. Administration and lecture satisfaction are also significantly associated with high stress. Students who are not satisfied with their administration and lectures are 9.479 times and 10.056 times more likely to experience high stress than those students who are satisfied with administration and lectures. Similarly, students who are not satisfied with teaching strategy are 1.209 times more likely to experience high stress compared to those who are satisfied. Moreover, students who are satisfied with their time management and do not have parental support in their study are 9.089 times and 16.376 times more likely to experience high stress. Furthermore, students who are not suffering from loneliness are 95.4% less likely to experience high stress compared to those who are suffering from loneliness. The remaining variables are insignificant at the 10% level of significance.

## Discussion

In the study, it was identified that 258 medical respondents from Shaheed Suhrawardy Medical College and Shaheed Monsur Ali Medical College, which comprises 67.9% of the sample, reported having moderate stress by the PSS-10 score. The perceived stress prevalence among medical students is high, like studies from Pakistan [37]and Portugal [38]. 9.5% and 22.6% of the respondents were identified as low and high-stressed students, respectively. The mean PSS score was 21.64, which is higher than those of medical students in Malaysia (19.5) [39], Korea (18.6) [40] and Thailand (13.5) [41] and lower than that of Saudi Arabia (28.5 ± 3.8) [42]. Table 6 shows the frequency distribution of the responses to the perceived stress scale by the medical students.

**Table 6:** Perceived stress scale (PSS-10) response (N = 380).

Variables	Never n (%)	Almost Never n (%)	Sometimes n (%)	Fairly Often n (%)	Very Often n (%)
Upset because something happened unexpectedly	23 (6.1%)	24 (6.3%)	186 (48.9%)	65 (17.1%)	82 (21.6%)
Unable to control important things in life	41 (10.8%)	39 (10.3%)	157 (41.3%)	73 (19.2%)	70 (18.4%)
Feeling nervous and stressed	20 (5.3%)	27 (7.1%)	147 (38.7%)	93 (24.5%)	93 (24.5%)
Confident in the ability to handle personal problems	59 (15.5%)	102 (26.8%)	148 (38.9%)	48 (12.6%)	23 (6.1%)
Felt that things were going your way	22 (5.8%)	78 (20.5%)	162 (42.6%)	74 (19.5%)	44 (11.6%)
Unable to cope with all the things to do	40 (10.5%)	57 (15.0%)	157 (41.3%)	65 (17.1%)	61 (16.1%)
Able to control irritations in life	42 (11.1%)	86 (22.6%)	173 (45.5%)	55 (14.5%)	24 (6.3%)
Feeling that you were on top of things	13 (3.4%)	35 (9.2%)	149 (39.2%)	91 (23.9%)	92 (24.2%)
Angered because of things outside of your control	35 (9.2%)	52 (13.7%)	164 (43.2%)	69 (18.2%)	60 (15.8%)
Feeling of difficulties piled up so high that you could not overcome them	54 (14.2%)	66 (17.4%)	152 (40.0%)	60 (15.8%)	48 (12.6%)

The perceived stress is significantly associated with several academic factors in bivariate analysis in the present study. Academic performance and anxiety regarding study and exam scores are significantly associated with the perceived stress of the medical students, which aligns with a study conducted in Germany regarding chronic stress in medical and dental students [43]. Academic curriculum and recreation time are significant academic stressors of the current study, which is in line with a study in India about perceived stress and stress sources of undergraduate medical students [44]. In Nepal, a study was conducted to identify the sources of stress among undergraduate medical students, which confirms that lecture satisfaction, lack of entertainment facilities, and exam pressure are significant sources of stress, and the current study also corroborates this finding [8]. Fear of uncertainty about the future and future choice cluelessness are important academic factors for perceived stress among medical students, which aligns with the study in Nigeria related to correlates of stress among medical students [45]. Class lecture satisfaction has a significant association with perceived stress, which aligns with a similar study conducted in Saudi Arabia [42].

Several psychological factors were also associated with the perceived stress. The most important source of stress was feeling loneliness in the current study, and it is also one of the prominent sources of stress that was identified in similar studies conducted in Pakistan [46] India [44]. Moreover, a study regarding perceived stress among undergraduate medical students was conducted in West Bengal, where it was found that peer conflict is significantly associated with perceived stress among students, and the current study is also consistent with this finding [47]. Additionally, time management skills and inability to interact with society are significantly associated with perceived stress according to the bivariate analysis.

Multivariate analysis was conducted where the perceived stress category was the dependent variable and the significant variables in bivariate analysis were the independent variables. Multinomial logistic regression identified the significant variables in multivariable analysis which are namely anxiety related to study and exams, difficulty in understanding context, fear of new living, administration and lecture satisfaction, teaching strategy satisfaction, time management, parental support in study, and feeling of loneliness. Students who are not anxious about their studies and exams are less likely to experience stress compared to those who experience anxiety. Moreover, students who face difficulty in understanding medical contexts suffer from moderate stress compared to those who understand the medical topics clearly. Students who are not afraid of new living are less likely to experience moderate and high stress compared to those who are afraid of new living. Lecture and Administration satisfaction are also significant predictors for stress of medical students and those who are not satisfied with lecture and administration are more likely to experience stress. Additionally, students who are not satisfied with the teaching strategy are more likely to experience stress compared to those who are satisfied with the teaching methods. Furthermore, students who are not satisfied with their time management are more likely to experience stress compared to those who are satisfied with their time management. Absence of parental support in study and loneliness are also key predictors and students are more likely to experience stress when their parents do not support their studies and their suffering from loneliness.

## Limitations of the study

The study sample was collected from two medical schools, and the entire analysis and results may not be generalizable to other medical schools in Bangladesh. Moreover, the data were self-reported, and hence the information was subject to reporting bias. Furthermore, the non-participation of several students might have impacted the sample size of the study. Despite these limitations, the study provides valuable insights into mental stress among medical students in Dhaka and offers an important foundation for future multi-institutional research.

## Conclusions

The cross-sectional study established the persistence of mental stress as a burden among medical students, as it identified that about 90% of the medical students as moderately and highly stressed. A substantial proportion of medical students were suffering from mental stress, mostly due to their pressures/demands. However, several psychological factors are also responsible in this regard. Considering the vulnerability of medical students towards mental stress and their potential contribution to future health systems, medical colleges and health authorities should investigate the matter by addressing their psychological needs and formulate effective strategies to reduce the burden of mental stress among medical students.

## Abbreviations

CI: Confidence Interval
RRR: Relative Risk Ratio
IRB: Institutional Review Board
NIPSOM: National Institute of Preventive and Social Medicine
PSS-10: Perceived Stress Scale-10
SPSS: Statistical Package for the Social Sciences
MBBS: Bachelor of Medicine and Bachelor of Surgery
